# Full Longitudinal Mediation Modeling Analysis of Frailty, Depression, and Cognitive Impairment

**DOI:** 10.1093/geroni/igaf122.3943

**Published:** 2025-12-31

**Authors:** Namhee Kim, Wongyeong Lee, Doukyoung Chon, Miji Kim, Chang Won Won

**Affiliations:** Yonsei University, Wonju, Republic of Korea; Yonsei University College of Nursing, Seoul, Republic of Korea; Yonsei University, Industry-Academic Cooperation Foundation of Future Medicine, Seoul, Republic of Korea; Kyung Hee University College of Medicine, Seoul, Republic of Korea; Kyung Hee University College of Medicine, Seoul, Republic of Korea

## Abstract

Frailty, depression, and cognitive impairment (CI) are interrelated geriatric syndromes that significantly impact the functioning and quality of life of older adults. In the context of a super-aging society, a clear understanding of their long-term interactions is essential for timely intervention and evidence-based policymaking. This study aimed to identify the directionality, mediating pathways, and critical time points for intervention by analyzing the dynamic interactions among them over time using the four-wave Korean Frailty and Aging Cohort Study (N = 1,377). Data were collected from a nationally representative sample of older adults in Korea from 2016 to 2023. Descriptive statistics characterized baseline levels and wave-specific changes. Pearson’s correlation coefficients assessed cross-sectional associations, while autoregressive and cross-lagged panel models evaluated temporal stability and causal directionality. Full Longitudinal Mediation Modeling (FLMM) explored potential mediation effects. All three variables showed significant temporal stability (β = .60–.75, p < .001). Depression in the initial stage significantly predicted increased frailty (β =.18, p < .01) and CI (β = –.12, p < .05) in subsequent waves, while the reverse effects were weak or insignificant. FLMM showed that depression mediated the effect of baseline cognitive function on future frailty, explaining approximately 35% of the total effect. Depression was identified as a significant mediator and longitudinal predictor of late-life physical and CI. Early screening and intervention for depressive symptoms can mitigate the progression to frailty and CI. These findings support integrated and timely strategies in community-based geriatric care and provide practical guidance for the effective allocation of preventive resources.

